# The Uniform System of Accounts

**Published:** 1907-03-02

**Authors:** 


### THE UNIFORM SYSTEM OF ACCOUNTS.

### NEW ANALYSIS LEDGER.

In accordance with the requirements of the King's Fund the modifications of this system of accounts, involving new books, will have come into force on January 1 last. It is very desirable to minimise the cost to the hospitals as much as possible. With this object in view, the Scientific Press (28 and 29 Southampton Street, W.C.) have prepared a new Analysis Ledger, ruled in accordance with, and adapted to the requirements of, the new index and classification recently adopted by King Edward's Hospital Fund, and the Hospital Saturday and Hospital Sunday Funds. The ledger is bound in half bazil, is printed and ruled on good paper, so as to make it in every way a serviceable book for hospital use. The price—32s. 6d.—is very moderate owing to the printing of the large number of copies, and should ensure a substantial sale.

